# Visfatin and Resveratrol Differentially Regulate the Expression of Thymidylate Synthase to Control the Sensitivity of Human Colorectal Cancer Cells to Capecitabine Cytotoxicity

**DOI:** 10.3390/life11121371

**Published:** 2021-12-09

**Authors:** Kuen-Lin Wu, Ko-Chao Lee, Chia-Kuang Yen, Cheng-Nan Chen, Shun-Fu Chang, Wen-Shih Huang

**Affiliations:** 1 Division of Colorectal Surgery, Department of Surgery, Kaohsiung Chang Gung Memorial Hospital, Kaohsiung 833, Taiwan; focus913@gmail.com (K.-L.W.); kmch4329@gmail.com (K.-C.L.); 2 Department of Food Science, National Chiayi University, Chiayi 600, Taiwan; Maple313@gmail.com; 3 Department of Biochemical Science and Technology, National Chiayi University, Chiayi 600, Taiwan; cnchen@mail.ncyu.edu.tw; 4 Department of Medical Research and Development, Chiayi Chang Gung Memorial Hospital, Chiayi 613, Taiwan; sfchang@cgmh.org.tw; 5 Graduate Institute of Clinical Medical Sciences, College of Medicine, Chang Gung University, Taoyuan 333, Taiwan; 6 Division of Colon and Rectal Surgery, Department of Surgery, Chiayi Chang Gung Memorial Hospital, Chiayi 613, Taiwan

**Keywords:** capecitabine, colorectal cancer, resveratrol, thymidylate synthase, visfatin

## Abstract

Colorectal cancer (CRC) is a highly lethal malignant cancer. Capecitabine, a 5-fluororacil (5-FU) derivate, is its first-line drug, but the resistance of CRC to capecitabine is still the most challenging factor for curing patients. It has been suggested that thymidylate synthase (TYMS) level might affect the capecitabine efficacy in CRC patients, but the mechanism still needs more elucidation. Obesity is a risk factor for CRC. Recently, a correlation between serum visfatin, an obesity-elicited adipokine, and CRC development has been found. Thus, the aim of present study is to examine the visfatin capacity in TYMS expression and in the development of capecitabine resistance of CRC. Moreover, an attractive natural component, i.e., resveratrol, has been proposed in anticancer therapy and has hence been examined in the present study to see its potential capacity in the alleviation of CRC resistance. Our results found that visfatin significantly reduces the CRC sensitivity to capecitabine by controlling the TYMS expression via p38 signaling and Sp1 transcription factor. Moreover, resveratrol could significantly alleviate the visfatin effect on capecitabine-treated CRC cells. These results provided new insights to understand the capecitabine susceptibility of CRC under a visfatin-containing environment and a possible therapeutic application of resveratrol in CRC patients with obesity.

## 1. Introduction

Colorectal cancer (CRC) has already been one of the commonest diagnosed and lethal malignant cancer types [1,2]. In the past several decades, because of the advance of theranostic drugs, tools, and instruments and the promotion of early CRC screening, the therapeutic and preventive efficacy of CRC has been adequately improved. However, it has been indicated that the incidence of CRC is still high and, even more, the age of patients suffering from CRC seems to be annually lowered [1,2]. The therapeutic strategy for CRC includes the single and combined treatment of chemotherapy, radiotherapy, and/or surgery. Currently, the biggest challenge for CRC therapy and further mortality improvement is the occurrence of resistance. [1,2,3]. Capecitabine is a fluoropyrimidines carbamate derivate, which is a prodrug and could be converted to the 5-fluororacil (5-FU). Capecitabine could be used as monotherapy and combined therapy with other drugs, e.g., oxaliplatin. After treatment, capecitabine would be directly converted to 5-FU at the cancer site. This is because the activated level of one of the main converting enzymes of capecitabine, i.e., thymidine phosphorylase, is much higher in cancer tissues [4,5]. This is also why the safety of oral capecitabine is better than intravenous 5-FU. However, since the end-product of capecitabine is 5-FU, it has been shown that the occurrence of resistance seems too similar to those involved in 5-FU [4,5]. Hence, a more detailed mechanism of study for 5-FU-based resistance is still an urgent need to further improve the clinical utility. Thymidylate synthase (TYMS) is an important enzyme in the nucleotide synthesis, which could convert deoxyuridine monophosphate (dUMP) to deoxythymidine monophosphate (dTMP) and could therefore be considered as a control target for capecitabine [6]. Increasing bench and clinical data has suggested that TYMS expression level might be an important determinant of cancer response to 5-FU-included chemotherapy. However, although the precise mechanism has not been clearly elucidated, a lot of evidence has noted that the TYMS level seems to be presented as a negative correlation to the efficacy of 5-FU-included chemotherapy [7,8]. Thus, TYMS has been proposed to potentially serve as a predictive biomarker of cell sensitivity and therapeutic target for 5-FU-included chemotherapy.

Obesity has been considered as one of the major causes of poor prognosis and recurrence of CRC [9,10,11]. This is because obesity would result in a systemic and chronic inflammatory environment by increasing the secretion of pro-inflammatory adipokines, e.g., visfatin, leptin, resistin, etc., and this condition would benefit the CRC development and attenuate the drug efficacy of patients [9,10,11]. Therefore, it has been proposed that the treatment and management of obesity is a real challenge in considering the therapeutic strategy of CRC patients. Recently, accumulating evidence has shown that the serum level of visfatin could be linked to the tumorigenicity of cancers, including CRC, gastric cancer, breast cancer, and brain cancer [12,13,14,15]. Moreover, it has been further shown that higher plasma visfatin level in patients could affect drug resistance of CRC and non-small cell lung cancer. Furthermore, the functional activities of visfatin could serve as an enzyme to convert nicotinamide into nicotinamide adenine dinucleotide or a secretory factor to initiate the signaling to affect cellular events. In addition, it has been shown that both functional roles of visfatin are associated with the CRC development [12,13,14,15]. Combined with these evidences, more and more studies have proposed the impact of visfatin in clinical application of CRC. However, its underlying mechanism still needs more precise elucidation.

Because of the low toxin and side effect, natural product compounds have attracted more and more attention and have gradually been applied in the treatment of various diseases, including cancer. Recently, accumulating clinical and bench studies have shown that increasing the dietary intake of polyphenols could effectively prevent the CRC development [16,17]. Resveratrol, a non-flavonoid polyphenol, has been extensively found in the skin of barriers, tomatoes, and grapes. It has been shown that resveratrol is becoming more and more prominent because of its great cancer-preventing and cancer-killing properties [18,19,20,21]. Moreover, accumulating data has also found that resveratrol could effectively recover the susceptibility of cancer cells to chemotherapy drugs, e.g., doxorubicin, by regulating certain signalings. Thus, in the present study, we would further determine if resveratrol could influence the visfatin effect on the capecitabine resistance development of CRC.

This study aimed to elucidate the possible visfatin capacity in the CRC susceptibility to capecitabine cytotoxicity and determine the possible prevented capacity of resveratrol in this process. It was shown that visfatin could upregulate the TYMS expression through p38 signaling and Sp-1 transcription factor and hence decrease the cytotoxicity of capecitabine in human DLD-1 CRC cells. Moreover, resveratrol could significantly recover this resistance-like phenomenon. These findings could contribute to new insights to understand the correlation between TYMS level and capecitabine resistance occurrence in visfatin-treated CRC cells and the potential improved application of resveratrol for capecitabine-based chemotherapy.

## 2. Materials and Methods

### 2.1. Materials

The materials used in the cell culture experiments were bought from Thermo (Waltham, MA, USA). The inhibitors, blocking kinase activity, were bought from Sigma (St. Louis, MO, USA), including inhibitors for ERK (PD98059), JNK (SP600125), and p38 (SB203580). The TYMS-, phosphor-p38-, p38-, and β-actin-specific antibodies (rabbit polyclone) were bought from Cell Signaling Technology (Beverly, MA, USA). The specific siRNAs for control, TYMS, and Sp1 were bought from Thermo (Waltham, MA, USA). All other materials used in this study were bought from Sigma (St. Louis, MO, USA).

### 2.2. Cell Culture

The human CRC cell line used in this study was DLD-1 CRC cell, which was bought from the Taiwan Food Industry Research and Development Institute (Hsinchu, Taiwan). DLD-1 CRC cells were cultured in Dulbecco’s Modified Eagle Medium, which was added with 10% fetal bovine serum and 1% antibiotics solution including penicillin and streptomycin, and then were placed in a 37 °C incubator supplemented with 5% carbon dioxide.

### 2.3. 3-(4,5-Dimethylthiazol-2-yl)-2,5-Diphenyltetrazolium Bromide (MTT) Assay

The viabilities of human DLD-1 CRC cells were determined by staining with MTT (0.5 mg/mL) and measuring their absorbances at 570 nm. The MTT-treated cells were incubated for 3 h and then further treated with dimethyl sulfoxide (DMSO) which could dissolve the formazan crystals. After that, the absorbance of the samples could be measured and analyzed.

### 2.4. Real-Time PCR

The treating cells were extracted by TRIzol reagent, and their RNAs were isolated and purified. After that, the mRNAs were further converted to the complementary DNA by reverse-transcription kit. The differential expression levels of various genes were analyzed by real-time PCR method with SYBR Green reagent bought from Applied Biosystems (Foster City, CA, USA) in a real-time PCR machine (Applied Biosystems, Foster City, CA, USA). Primers: TYMS (+), 5′-CCTCT GCTGA CAACC AAACG-3′; TYMS (-), 5′-GAAGA CAGCT CTTTA GCATT TG-3′; 18S rRNA (+), 5′-CGGCG ACGAC CCATT CGAAC-3′, and 18S rRNA (-), 5′-GAATC GAACC CTGAT TCCCC GTC-3′. Quantification was performed using the 2^−ΔΔ^*^C^*^t^ method.

### 2.5. Western Blot

The proteins of treating cells were extracted by a commercial lysis buffer (Millipore, Darmstadt, Germany) with the protease/phosphatase inhibitor cocktail (Roche, Basel, Switzerland). The concentrations of total proteins were analyzed by using the Bio-Rad protein quantification reagent (Hercules, CA, USA). The expression and phosphorylation levels of proteins were analyzed by the SDS-PAGE with the specific antibodies and the chemiluminescent system (Bio-Rad, Hercules, CA, USA).

### 2.6. Activity Analysis of Transcription Factor

The nuclear proteins of human DLD-1 CRC cells were extracted by the extraction buffer (Panomics, Redwood City, CA), and then their concentrations were analyzed by using the Bio-Rad protein quantification reagent (Hercules, CA, USA). The activity of Sp1 transcription factor was determined by using the commercial ELISA kits (Panomics, Redwood City, CA, USA).

### 2.7. Trypan Blue Dye Exclusion Assay

It has been shown that trypan blue could penetrate the dead cells but exclude the living cells. Therefore, after experiments, the cells were collected and stained with trypan blue reagent, and then the stained cells were counted on a hemocytometer and the proportion of dead cells was determined. Dead cells were counted: trypan blue-penetrated cell ratio (%) = (number of stained cells/total number of cells) × 100.

### 2.8. Statistical Analysis

The statistical form was shown as the mean ± standard error of the mean. The statistics of experimental data were calculated by an independent Student’s t-test for two groups per experiment and analysis of variance followed by Scheffe’s test for multiple comparisons. The *p* values smaller than 0.05 could be shown as statistically significant. The experiments in the present study must be compared with three independent repetitions.

## 3. Results

### 3.1. Visfatin Increases the TYMS Expression in Human DLD-1 CRC Cells

**Figure 1:**
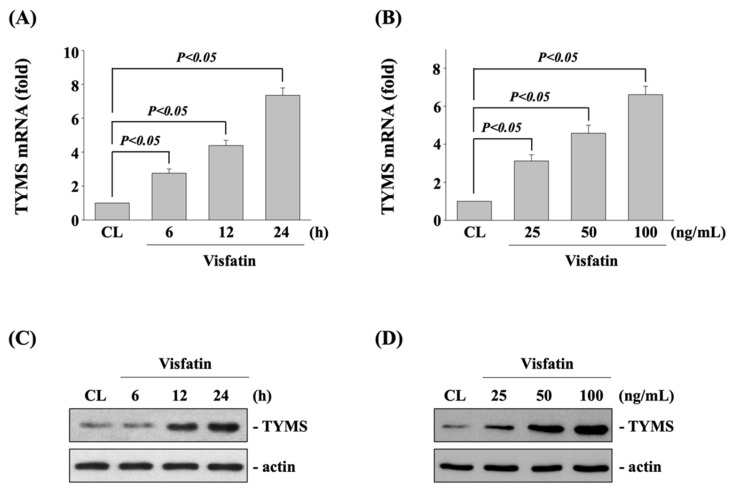
Visfatin increases the TYMS expression in human DLD-1 CRC cells. Human DLD-1 CRC cells were kept as control (CL) or (**A**,**C**) treated with visfatin (50 ng/mL) for 6, 12, and 24 h or (**B**,**D**) treated with different doses of visfatin (25, 50, and 100 ng/mL) for 12 h. The mRNA (**A**,**B**) and protein (**C**,**D**) expressions of TYMS of were examined by real-time PCR and Western blot, respectively. Data in (**A**,**B**) were shown as mean ± SEM from three independent experiments. Results in (**C**,**D**) were representative of three independent experiments with similar results. *p* < 0.05 was defined as statistically significant.

Human DLD-1 CRC cells were kept as control or treated with visfatin (25, 50, and 100 ng/mL) for 6, 12, and 24 h and then the mRNA and protein expression of TYMS were examined by real-time PCR and Western blot, respectively. It was shown that treating cells with visfatin significantly increases the expressions of TYMS mRNA (Figure 1A,B) and protein (Figure 1C,D) in time-dependent (50 ng/mL for 6, 12, and 24 h) and dose-dependent (25, 50 and 100 ng/mL for 12 h) manners compared with untreated control cells.

### 3.2. TYMS Upregulation of Visfatin Induction Attenuates the Sensitivity of Human DLD-1 CRC Cells to Capecitabine Cytotoxicity

**Figure 2:**
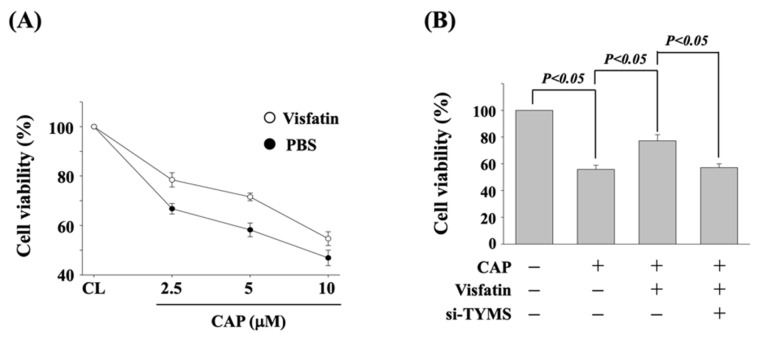
TYMS upregulation of visfatin induction attenuates the sensitivity of human DLD-1 CRC cells to capecitabine cytotoxicity. (**A**) Human DLD-1 CRC cells were pretreated with vehicle (PBS) or visfatin (50 ng/mL) for 1 h and then kept as control or treated with capecitabine (2.5, 5, and 10 μM) for 24 h. (**B**) Human DLD-1 CRC cells were transfected with control- or TYMS-specific siRNA (si-TYMS) for 48 h and then pretreated with vehicle (PBS) or visfatin (50 ng/mL) for 1 h and further kept as control or treated with capecitabine (CAP, 5 μM) for 24 h. (**A**,**B**) The viability of treated cells was examined by MTT assay. Data were shown as mean ± SEM from three independent experiments. *p* < 0.05 was indicated as statistically significant.

To determine if TYMS upregulation of visfatin induction influences the cytotoxicity of capecitabine in human DLD-1 CRC cells, cells were pretreated with vehicle (PBS) or visfatin (50 ng/mL) for 1 h and then kept as control or treated with capecitabine (2.5, 5, and 10 μM) for 24 h. The viability of treated cells was examined by MTT assay. Cells co-treated with capecitabine and visfatin increased the cell viability of human DLD-1 CRC cells as compared with the cells treated with capecitabine alone (Figure 2A). Moreover, human DLD-1 CRC cells were transfected with control- or TYMS-specific siRNA for 48 h to knockdown the respective gene expression. Cells were further pretreated with vehicle (PBS) or visfatin (50 ng/mL) for 1 h and kept as control or treated with capecitabine (5 μM) for 24 h. The viability of treated cells was examined by MTT assay. It was shown that the gene knockdown of TYMS recovers the visfatin-attenuated cytotoxicity of capecitabine in human DLD-1 CRC cells (Figure 2B).

### 3.3. p38 Signaling Regulates the Visfatin Effect on TYMS Upregulation and Subsequent Capecitabine-Induced Death in Human DLD-1 CRC Cells

**Figure 3:**
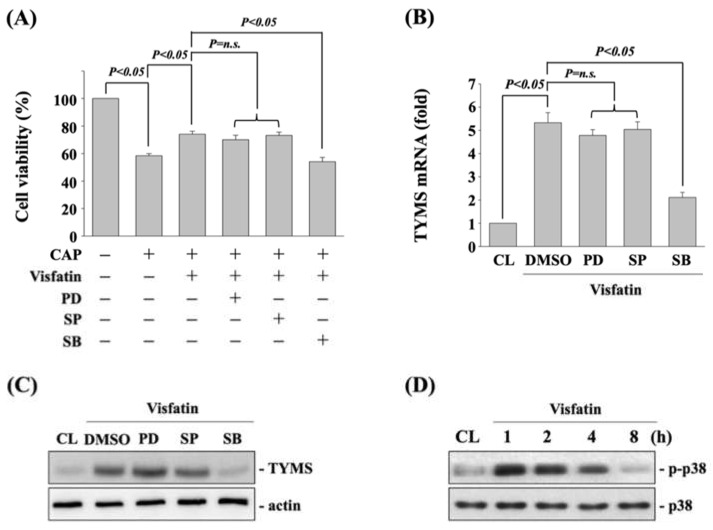
p38 signaling regulates the visfatin effect on TYMS upregulation and subsequent capecitabine-induced death in human DLD-1 CRC cells. (**A**) Human DLD-1 CRC cells were pretreated with vehicle (DMSO) or inhibitors of ERK1/2 (PD98059, 25 μM), JNK (SP600125, 20 μM), and p38 (SB203580, 10 μM) for 30 min and then treated with vehicle (PBS) or visfatin (50 ng/mL) for 1 h and further kept as control or treated with capecitabine (CAP, 5 μM) for 24 h. The viability of treated cells was examined by MTT assay. (**B**,**C**) Human DLD-1 CRC cells were pretreated with vehicle (DMSO) or inhibitors of ERK1/2 (PD98059, 25 μM), JNK (SP600125, 20 μM), and p38 (SB203580, 10 μM) for 30 min and then treated with visfatin (50 ng/mL) for 12 h. The mRNA (**B**) and protein (**C**) expressions of TYMS were examined by real-time PCR and Western blot, respectively. (**D**) Human DLD-1 CRC cells were kept as control (CL) or treated with visfatin (50 ng/mL) for 1, 2, 4, and 8 h, and then the phosphorylation of p38 kinase was examined by Western blot. Data in (**A**,**B**) were shown as mean ± SEM from three independent experiments. Results in (**C**,**D**) were representative of three independent experiments with similar results. *p* < 0.05 was indicated as statistically significant. n.s. is the abbreviation of no significance.

To determine if MAPK signaling, including ERK1/2, JNK, and p38 kinases, regulates the visfatin effect on TYMS upregulation and subsequent capecitabine-induced death in human DLD-1 CRC cells, cells were pretreated with vehicle (DMSO) or inhibitors of ERK1/2 (PD98059, 25 μM), JNK (SP600125, 20 μM), and p38 (SB203580, 10 μM) for 30 min. After that, cells were further treated with vehicle (PBS) or visfatin (50 ng/mL) for 1 h and then kept as control or treated with capecitabine (5 μM) for 24 h. The viability of treated cells was examined by MTT assay. It was shown that cells pretreated with p38 inhibitor recover the visfatin effect on capecitabine-induced death in human DLD-1 CRC cells (Figure 3A). Moreover, p38 signaling inhibition also attenuated the visfatin-increased mRNA (Figure 3B) and protein (Figure 3C) expressions of TYMS in human DLD-1 CRC cells. Cells treated with visfatin (50 ng/mL) also resulted in a significant increase in the phosphorylation of p38 kinase within 1 h and persisted for 4 h in human DLD-1 CRC cells compared with the untreated control (Figure 3D).

### 3.4. Sp1 Transcription Factor Regulates the TYMS Expression and Subsequent Capecitabine-Induced Death in Visfatin-Treated Human DLD-1 CRC Cells

**Figure 4:**
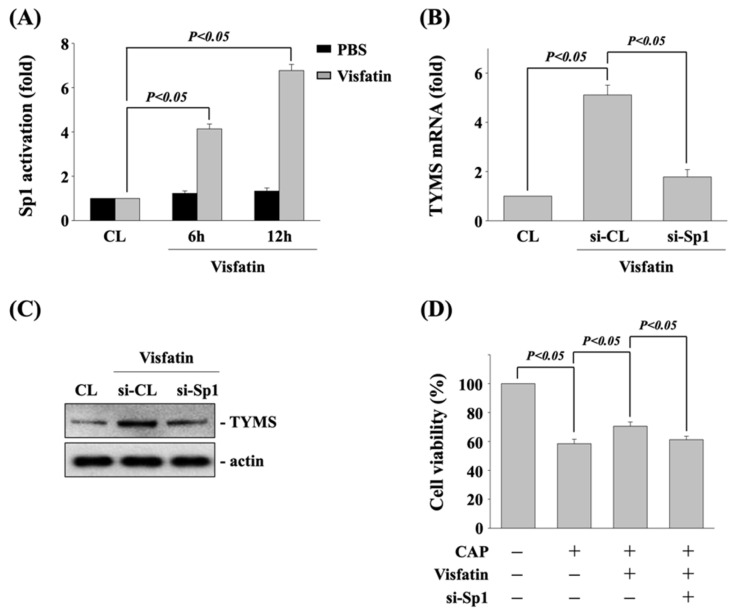
Sp1 transcription factor regulates the TYMS expression and subsequent capecitabine-induced death in visfatin-treated human DLD-1 CRC cells. (**A**) Human DLD-1 CRC cells were kept as control or treated with vehicle (PBS) or visfatin (50 ng/mL) for 6 and 12 h, and then the activity of Sp1 transcription factor was determined by transcription factor ELISA assay. (**B**,**C**) Human DLD-1 CRC cells were transfected with control- or Sp1-specific siRNA for 48 h and then kept as control or treated with visfatin for 12 h, and then the expressions of (**B**) mRNA and (**C**) protein of TYMS were examined by real-time PCR and Western blot, respectively. (**D**) Human DLD-1 CRC cells were transfected with control- or TYMS-specific siRNA for 48 h and then pretreated with vehicle (PBS) or visfatin (50 ng/mL) for 1 h and further kept as control or treated with capecitabine (CAP, 5 μM) for 24 h. The viability of treated cells was examined by MTT assay. Data in (**A**,**B**,**D**) were shown as mean ± SEM from three independent experiments. Results in (**C**) were representative of three independent experiments with similar results. *p* < 0.05 was indicated as statistically significant.

Sp1 could be one of transcription factors for TYMS expression in accordance with various stimulators [22]. Hence, we determine if Sp1 transcription factor regulates the visfatin effect on TYMS expression and subsequent capecitabine-induced death in human DLD-1 CRC cells. Cells were kept as control or treated with vehicle (PBS) or visfatin (50 ng/mL) for 6 and 12 h, and then the activity of Sp1 transcription factor was determined by transcription factor ELISA assay. Human DLD-1 CRC cells stimulated with visfatin activated Sp1 transcription factor in a time-dependent manner compared with the untreated control and PBS-treated cells (Figure 4A). Next, cells were kept as control or transfected with control- or Sp1 transcription factor-specific siRNA to knockdown the respective gene expression. After that, the transfected cells were further treated with visfatin (50 ng/mL) for 12 h, and then the expressions of TYMS mRNA and protein were determined by real-time PCR and Western blot, respectively. It was shown that Sp1 gene knockdown significantly inhibits the visfatin-increased TYMS mRNA (Figure 4B) and protein (Figure 4C) expression. Moreover, the transfected cells were also further pretreated with vehicle (PBS) or visfatin (50 ng/mL) for 1 h and kept as control or treated with capecitabine (5 μM) for 24 h. The viability of treated cells was examined by MTT assay. It was shown that Sp1 gene knockdown also recovers the visfatin effect on capecitabine-induced death in human DLD-1 CRC cells (Figure 4D).

### 3.5. Resveratrol Attenuates the Visfatin Effect on Capecitabine-Induced Death in Human DLD-1 CRC Cells

**Figure 5:**
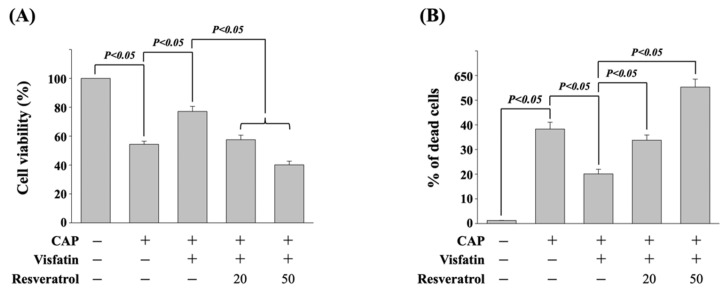
Resveratrol attenuates the visfatin effect on capecitabine-induced death in human DLD-1 CRC cells. (**A**,**B**) Human DLD-1 CRC cells were pretreated with vehicle (ethanol) or resveratrol (20 and 50 μM) for 1 h and then treated with vehicle (PBS) or visfatin (50 ng/mL) for 1 h and further kept as control or treated with capecitabine (CAP, 5 μM) for 24 h. The viability of treated cells was examined by MTT assay (**A**) and trypan blue dye exclusion assay (**B**). The percentage of trypan blue-positive cells represented the population of dead cells. Data in (**A**,**B**) were shown as mean ± SEM from three independent experiments. *p* < 0.05 was indicated as statistically significant.

It has been revealed that resveratrol could be a potential natural compound in cancer therapy [18]. Hence, next, we determined if resveratrol could influence the visfatin effect on reducing the cytotoxicity of capecitabine in human DLD-1 CRC cells. Cells were pretreated with vehicle (ethanol) or resveratrol (20 and 50 μM) for 1 h. After that, cells were further treated with vehicle (PBS) or visfatin (50 ng/mL) for 1 h and then kept as control or treated with capecitabine (5 μM) for 24 h. The viability of treated cells was examined by MTT assay and trypan blue dye exclusion assay. It was shown that resveratrol significantly attenuates the visfatin effect on reducing the cytotoxicity of capecitabine in human DLD-1 CRC cells in a dose-dependent manner, which resulted in a recovery in the visfatin-increased cell viability (Figure 5A) and decreased cell death (Figure 5B).

### 3.6. Resveratrol Inhibits the Visfatin Effect on TYMS Expression, p38 Phosphorylation, and Sp1 Transcriptional Activation

**Figure 6:**
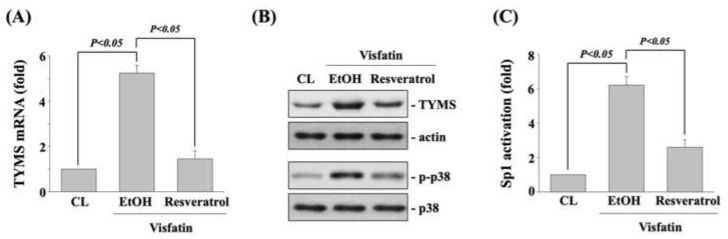
Resveratrol inhibits the visfatin effect on TYMS expression, p38 phosphorylation, and Sp1 transcriptional activation. Human DLD-1 CRC cells were pretreated with vehicle (ethanol) or resveratrol (50 μM) for 1 h and then treated with vehicle (PBS) or visfatin (50 ng/mL) for (**A**–**C**) 12 h (TYMS expression and Sp1 transcriptional activation) and for (**B**) 1 h (p38 phosphorylation). (**A**,**B**) The mRNA (real-time PCR) and protein (Western blot) expressions of TYMS, (**B**) p38 phosphorylation (Western blot), and (**C**) SP1 transcriptional activity (transcription factor ELISA assay) in human DLD-1 CRC cells were determined. Data in (**A**,**C**) were shown as mean ± SEM from three independent experiments. Results in (**B**) were representative of three independent experiments with similar results. *p* < 0.05 was indicated as statistically significant.

Finally, human DLD-1 CRC cells were pretreated with vehicle (ethanol) or resveratrol (50 μM) for 1 h and then kept as control or treated with visfatin (50 ng/mL) for 1 h (p38 phosphorylation) and 12 h (TYMS expression and Sp1 transcriptional activation). The mRNA (real-time PCR) and protein (Western blot) expressions of TYMS, p38 phosphorylation (Western blot), and SP1 transcriptional activity (transcription factor ELISA assay) in human DLD-1 CRC cells were determined. Treating cells with visfatin significantly increased the TYMS mRNA (Figure 6A) and protein (upper panel in Figure 6B) expression, p38 phosphorylation (lower panel in Figure 6B), and Sp1 transcriptional activation (Figure 6C) compared to the untreated control. However, pretreating cells with resveratrol significantly inhibited these visfatin effects on human DLD-1 CRC cells.

## 4. Discussion

The resistance development to chemotherapy severely limits the treating outcomes and remains a major challenge faced by clinicians [23]. Capecitabine is a prodrug of fluorouracil and has been developed as a cancer-selective fluoropyrimidine carbamate to achieve high cytotoxic concentration of fluorouracil at cancer sites [4]. However, the occurrence of capecitabine resistance is still the main limiting factor hindering the patients’ treatment efficacy. Thus, studying the mechanism of capecitabine resistance in cancer cells can provide the opportunity to fundamentally improve the overall survival rate. This study was to examine the capacity of TYMS expression level in CRC cells in response to capecitabine cytotoxicity under obesity-visfatin stimulation and try to find a potential enhancer capacity of resveratrol in anti-CRC progression. Our results present several important respects: (1) Expression of TYMS in CRC cells could be induced while visfatin treatment and this induction could accordingly decrease the cytotoxicity of capecitabine in human DLD-1 CRC cells. (2) p38 signaling and Sp1 transcription factor controlled the visfatin effect on TYMS expression and subsequent capecitabine-induced cell death. (3) Resveratrol could effectively improve the visfatin signaling and effect on capecitabine-induced death in human DLD-1 CRC cells. Thus, these results have explained a reversed drug resistance by which TYMS expression level could be regulated to influence the CRC sensitivity to capecitabine.

The crucial capacity of TYMS in DNA biosynthesis and tumorigenesis has been evidenced, which allows TYMS to be considered as a great target for 5-FU-included chemotherapy [6,7,8]. This is because the property of high binding affinity of TYMS with dUMP also allows a tight association between TYMS and FdUMP after 5-FU-included chemotherapy. The TYMS/FdUMP-associated complex would further compete the binding of normal dUMP and might perturb the levels of others deoxynucleotides. Both of the conditions finally lead to the DNA replication stop and lethal DNA lesion, and these are the underlying anticancer mechanisms of 5-FU-included chemotherapy. However, scientists and clinicians have further found that 5-FU treatment could also acutely result in the abnormal expression of TYMS in cancer cells. Moreover, this high expression level of TYMS has been found to be correlated with the development of poor 5-FU sensitivity in cancer cells [24,25,26]. In fact, in the clinical evidence, in the patients with metastatic or advanced CRC, lower TYMS expression level in cancer tissues is indeed a better outcome to 5-FU-included chemotherapy compared with the higher expression level [27,28]. This previous evidence supports our present findings that TYMS upregulation would affect the efficiency of capecitabine-induced CRC cell death. Moreover, our data about visfatin-increased TYMS expression level through p38 signaling and Sp1 transcription factor further proposed a possible role and mechanism of obesity in CRC patients to initiate the resistance of 5-FU-included chemotherapy through regulating the TYMS expression level, and this should be considered in the future therapeutic strategy.

There is no doubt that the drug sensitivity of cells is determined by complex mechanisms. Much evidence from bench and clinical studies has shown that visfatin expression level could regulate the progression of CRC and others various cancers [12]. It has been found that the increased serum level of visfatin might be a major cause leading to a more malignant phenotype in CRC cells and patients [12,29]. Therefore, visfatin could have a high impact to be considered as a therapeutic target in the clinical treatment of CRC patients. In this study, we showed that visfatin-induced TYMS expression, which results in decreased sensitivity to capecitabine-induced cytotoxicity, might be a significant contributor to the occurrence of resistance in CRC cells. Moreover, the inhibition of visfatin-induced TYMS by resveratrol could recover the decreased CRC sensitivity to capecitabine cytotoxicity and hence recover the rate of cell death. Several studies have suggested that visfatin could be related to the development of cancer resistance. It has been revealed that visfatin could reduce the sensitivity of non-small cell lung cancer cells to doxorubicin by activating the Akt signaling [13]. Moreover, in CRC, (i) plasma level of visfatin could be used as a prognostic factor of poor response to 5-FU-included chemotherapy because a higher level of visfatin is observed in patients undergoing disease progression compared with the partial remission and stable disease groups [12], and (ii) inhibition of endogenous visfatin expression could downregulate the doxorubicin resistance of CRC cells [30]. Our present study also showed that visfatin-increased TYMS expression is controlled by p38 signaling and Sp1 transcription factor. p38 signaling is a well-defined pathway in regulating cell survival and death and could also have a capacity in DNA damage response. Previous studies have also shown the correlation between p38 activation and the resistance development of cancer cells [31,32]. Sp1 transcription factor has also been found to have an impact in controlling the transcription of TYMS [22]. Therefore, the results of this study suggested that visfatin-activated p38 signaling and Sp1 transcription factor are crucial elicitors to promote drug resistance of CRC cells through upregulating the TYMS expression.

Considering natural product components as drug sources is a rapidly interest-increasing field. Resveratrol could be a great example because of its pharmacological efficacy in anti-inflammation, anti-oxidation, lipid-lowering, hypoglycemic, and cancer inhibition [33]. The anticancer activity of resveratrol has been found in breast cancer, head and neck squamous carcinoma, liver cancer, bladder cancer, prostate cancer, and CRC [18,34]. Moreover, it has also been indicated that the combined therapy of resveratrol with clinical chemotherapy, e.g., 5-FU and oxaliplatin, could effectively re-sensitize cancer cells to those chemotherapeutic agents, which also means resveratrol could potentially lower the occurrence of resistance [34]. Furthermore, it has been revealed that the anticancer efficacy of resveratrol could be due to its inflammation- and oxidative stress-inhibiting properties. In the present study, resveratrol was shown to decrease the visfatin effect on lowering the sensitivity of CRC cells to capecitabine. As described earlier, visfatin is a well-defined inflammation-stimulating adipokine derived from obesity environment. Hence, one of the limitations of our study is that we did not further assay whether the possibility of resistance-lowering event of resveratrol is through blocking the inflammatory and oxidative responses of visfatin in CRC cells. Moreover, our data could propose that the combined therapy of resveratrol with 5-FU-included chemotherapy might be a great strategy for treating the CRC patients with obesity.

## 5. Conclusions

Our present study has indicated that visfatin could attenuate the sensitivity of CRC to capecitabine cytotoxicity through controlling the TYMS expression via p38 signaling and Sp1 transcription factor. Moreover, resveratrol could be considered to be used in combined therapy with 5-FU-included chemotherapy to alleviate the visfatin effect. These results provided new insights to understand the capecitabine susceptibility of CRC under visfatin-containing environment and a possible therapeutic application of resveratrol in CRC patients with obesity.

## Data Availability

The original images of Western blot are shown in Supplementary Materials Figure S1.

## Supplementary Materials

The following are available online at https://www.mdpi.com/article/10.3390/life11121371/s1, Figure S1: Gel images of Western blot results (Figure 1, Figure 3, Figure 4, and Figure 6).

## References

[1] Sung J.J.Y., Chiu H.M., Jung K.W., Jun J.K., Sekiguchi M., Matsuda T., Kyaw M.H. (2019). Increasing Trend in Young-Onset Colorectal Cancer in Asia: More Cancers in Men and More Rectal Cancers. Am. J. Gastroenterol..

[2] Vodenkova S., Buchler T., Cervena K., Veskrnova V., Vodicka P., Vymetalkova V. (2020). 5-fluorouracil and other fluoropyrimidines in colorectal cancer: Past, present and future. Pharmacol. Ther..

[3] Sánchez-Hidalgo J.M., Rodríguez-Ortiz L., Arjona-Sánchez Á., Rufián-Peña S., Casado-Adam Á., Cosano-Álvarez A., Briceño-Delgado J. (2019). Colorectal peritoneal metastases: Optimal management review. World J. Gastroenterol..

[4] Furukawa T., Tabata S., Yamamoto M., Kawahara K., Shinsato Y., Minami K., Shimokawa M., Akiyama S.I. (2018). Thymidine phosphorylase in cancer aggressiveness and chemoresistance. Pharmacol. Res..

[5] Chong G., Dickson J.L.B., Cunningham D., Norman A.R., Rao S., Hill M.E., Price T.J., Oates J., Tebbutt N. (2005). Capecitabine and mitomycin C as third-line therapy for patients with metastatic colorectal cancer resistant to fluorouracil and irinotecan. Br. J. Cancer.

[6] Botticelli A., Scagnoli S., Roberto M., Lionetto L., Cerbelli B., Simmaco M., Marchetti P. (2020). 5-Fluorouracil degradation rate as a predictive biomarker of toxicity in breast cancer patients treated with capecitabine. J. Oncol. Pharm. Pract..

[7] Blondy S., David V., Verdier M., Mathonnet M., Perraud A., Christou N. (2020). 5-Fluorouracil resistance mechanisms in colorectal cancer: From classical pathways to promising processes. Cancer Sci..

[8] Sakatani A., Sonohara F., Goel A. (2019). Melatonin-mediated downregulation of thymidylate synthase as a novel mechanism for overcoming 5-fluorouracil associated chemoresistance in colorectal cancer cells. Carcinogenesis.

[9] Moodi M., Tavakoli T., Tahergorabi Z. (2021). Crossroad between obesity and gastrointestinal cancers: A review of molecular mechanisms and interventions. Int. J. Prev. Med..

[10] Pu Z., Chen S. (2021). Targeting adipokines in obesity-related tumors. Front. Oncol..

[11] Quail D.F., Dannenberg A.J. (2019). The obese adipose tissue microenvironment in cancer development and progression. Nat. Rev. Endocrinol..

[12] Lin T.C. (2019). The role of visfatin in cancer proliferation, angiogenesis, metastasis, drug resistance and clinical prognosis. Cancer Manag. Res..

[13] Cao Z., Liang N., Yang H., Li S. (2017). Visfatin mediates doxorubicin resistance in human non-small-cell lung cancer via Akt-mediated up-regulation of ABCC1. Cell Prolif..

[14] Ji C., Cong R., Wang Y., Wang Y., Zhang Q., Zhou X., Xing Q., Song N. (2019). Relationship between NAMPT/PBEF/visfatin and prognosis of patients with malignant tumors: A systematic review and meta-analysis. Ann. Transl. Med..

[15] Chen M., Wang Y., Li Y., Zhao L., Ye S., Wang S., Yu C., Xie H. (2016). Association of plasma visfatin with risk of colorectal cancer: An observational study of Chinese patients. Asia Pac. J. Clin. Oncol..

[16] Alam M.N., Almoyad M., Huq F. (2018). Polyphenols in Colorectal Cancer: Current State of Knowledge including Clinical Trials and Molecular Mechanism of Action. Biomed Res. Int..

[17] Cueva C., Silva M., Pinillos I., Bartolomé B., Moreno-Arribas M.V. (2020). Interplay between Dietary Polyphenols and Oral and Gut Microbiota in the Development of Colorectal Cancer. Nutrients.

[18] Honari M., Shafabakhsh R., Reiter R.J., Mirzaei H., Asemi Z. (2019). Resveratrol is a promising agent for colorectal cancer prevention and treatment: Focus on molecular mechanisms. Cancer Cell Int..

[19] Arabzadeh A., Mortezazadeh T., Aryafar T., Gharepapagh E., Majdaeen M., Farhood B. (2021). Therapeutic potentials of resveratrol in combination with radiotherapy and chemotherapy during glioblastoma treatment: A mechanistic review. Cancer Cell Int..

[20] Xiao Q., Zhu W., Feng W., Lee S.S., Leung A.W., Shen J., Gao L., Xu C. (2019). A Review of Resveratrol as a Potent Chemoprotective and Synergistic Agent in Cancer Chemotherapy. Front. Pharmacol..

[21] Huang L., Zhang S., Zhou J., Li X. (2019). Effect of resveratrol on drug resistance in colon cancer chemotherapy. RSC Adv..

[22] Dong S., Lester L., Johnson L.F. (2000). Transcriptional control elements and complex initiation pattern of the TATA-less bidirectional human thymidylate synthase promoter. J. Cell Biochem..

[23] Bukowski K., Kciuk M., Kontek R. (2020). Mechanisms of Multidrug Resistance in Cancer Chemotherapy. Int. J. Mol. Sci..

[24] Varghese V., Magnani L., Harada-Shoji N., Mauri F., Szydlo R.M., Yao S., Lam E.W., Kenny L.M. (2019). FOXM1 modulates 5-FU resistance in colorectal cancer through regulating TYMS expression. Sci. Rep..

[25] Marangoni E., Laurent C., Coussy F., El-Botty R., Château-Joubert S., Servely J.L., de Plater L., Assayag F., Dahmani A., Montaudon E. (2018). Capecitabine Efficacy is Correlated with TYMP and RB1 Expression in PDX Established from Triple-Negative Breast Cancers. Clin. Cancer Res..

[26] Terranova-Barberio M., Roca M.S., Zotti A.I., Leone A., Bruzzese F., Vitagliano C., Scogliamiglio G., Russo D., D’Angelo G., Franco R. (2016). Valproic acid potentiates the anticancer activity of capecitabine in vitro and in vivo in breast cancer models via induction of thymidine phosphorylase expression. Oncotarget.

[27] Jiang H., Li B., Wang F., Ma C., Hao T. (2019). Expression of ERCC1 and TYMS in colorectal cancer patients and the predictive value of chemotherapy efficacy. Oncol. Lett..

[28] Abdallah E.A., Fanelli M.F., Buim M.E., Machado Netto M.C., Gasparini Junior J.L., Silva V.S.E., Dettino A.L., Mingues N.B., Romero J.V., Ocea L.M. (2015). Thymidylate synthase expression in circulating tumor cells: A new tool to predict 5-fluorouracil resistance in metastatic colorectal cancer patients. Int. J. Cancer.

[29] Yang J., Zhang K., Song H., Wu M., Li J., Yong Z., Jiang S., Kuang X., Zhang T. (2016). Visfatin is involved in promotion of colorectal carcinoma malignancy through an inducing EMT mechanism. Oncotarget.

[30] Yan X., Zhao J., Zhang R. (2017). Visfatin mediates doxorubicin resistance in human colorectal cancer cells via up regulation of multidrug resistance 1 (MDR1). Cancer Chemother. Pharmacol..

[31] Hu H., Han T., Zhuo M., Wu L.L., Yuan C., Wu L., Lei W., Jiao F., Wang L.W. (2017). Elevated COX-2 Expression Promotes Angiogenesis Through EGFR/p38-MAPK/Sp1-Dependent Signalling in Pancreatic Cancer. Sci. Rep..

[32] Fang H., Ma W., Guo X., Wang J. (2021). PTPN6 promotes chemosensitivity of colorectal cancer cells via inhibiting the SP1/MAPK signalling pathway. Cell Biochem. Funct..

[33] Koushki M., Amiri-Dashatan N., Ahmadi N., Abbaszadeh H.A., Rezaei-Tavirani M. (2018). Resveratrol: A miraculous natural compound for diseases treatment. Food Sci. Nutr..

[34] Li D., Wang G., Jin G., Yao K., Zhao Z., Bie L., Guo Y., Li N., Deng W., Chen X. (2019). Resveratrol suppresses colon cancer growth by targeting the AKT/STAT3 signaling pathway. Int. J. Mol. Med..

