# Supplementary material for: Visfatin and Resveratrol Differentially Regulate the Expression of Thymidylate Synthase to Control the Sensitivity of Human Colorectal Cancer Cells to Capecitabine Cytotoxicity
Source: Life (Basel). 2021 Dec 9;11(12):1371. doi: 10.3390/life11121371 (PMC8704879; doi:10.3390/life11121371)
Supplement: Supplementary file 1 [file life-11-01371-s001.zip › Visfatin-Resveratrol-Capecitabine-Raw data.pdf]

Figure 1C

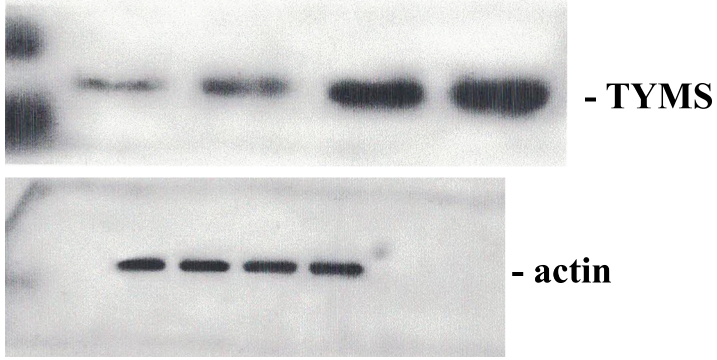

Figure 3C

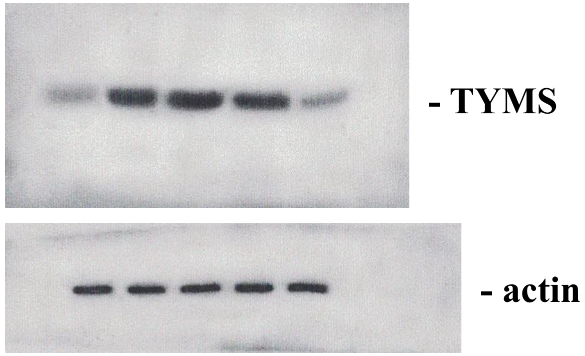

Figure 4C

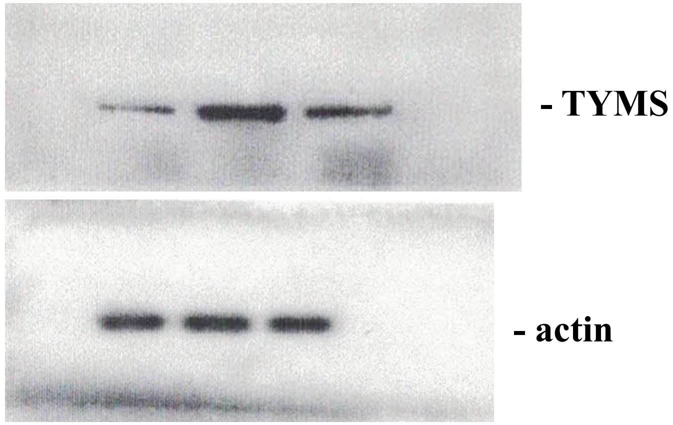

Figure 1D

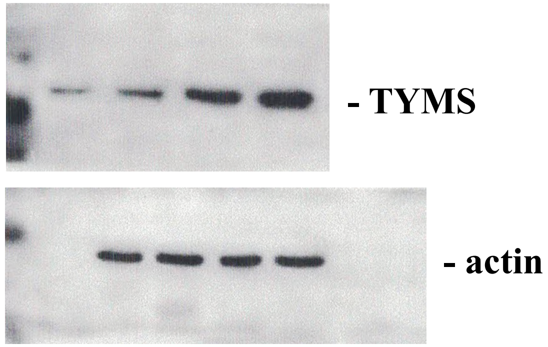

Figure 3D

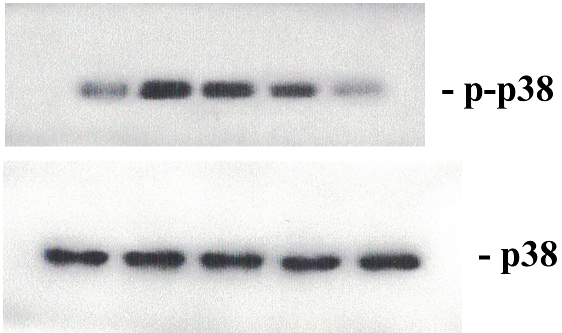

Figure 6B

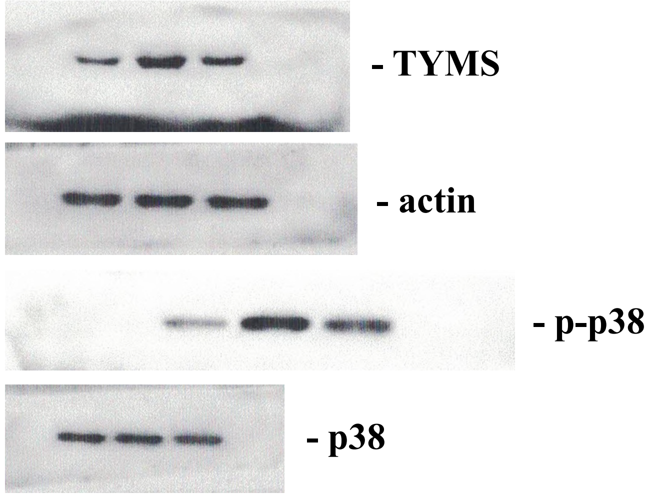

Figure S1: Gel images of Western blot in Figures 1, 3, 4, and 6.
